# Epstein-Barr virus variation in people living with human immunodeficiency virus in southeastern China

**DOI:** 10.1186/s12985-023-02078-z

**Published:** 2023-05-31

**Authors:** Zhikai Wan, Ying Chen, Jiangjin Hui, Yongzheng Guo, Xiaorong Peng, Mengyan Wang, Caiqin Hu, Yirui Xie, Junwei Su, Ying Huang, Xiaoke Xu, Yan Xu, Biao Zhu

**Affiliations:** The Department of Infectious Diseases, State Key Laboratory for Diagnosis and Treatment of Infectious Diseases, National Clinical Research Center for Infectious Diseases, Collaborative Innovation Center for Diagnosis and Treatment of Infectious Diseases, the First Affiliated Hospital, School of Medicine, Zhejiang University, 79 Qing Chun Road, Hangzhou, 310006 China

**Keywords:** EBV, HIV, Subtype, Viral load, LMP-1, Variation, China

## Abstract

**Background:**

Patients infected with HIV are at high risk of developing Epstein-Barr Virus (EBV)-related diseases. The genotype and viral biological behavior of EBV infection in patients with human immunodeficiency virus-1 (HIV) in China remain unclear. This study analyzed the characteristics of EBV in patients infected with HIV in southeastern China.

**Methods:**

A total of 162 HIV-infected patients and 52 patients without HIV were enrolled in this study. EBV viral load in blood was determined by fluorescence quantitative PCR. EBV typing was performed using saliva according to polymorphisms in the EBNA3C region. EBV LMP-1 carboxy terminus (C-ter) was sequenced, and compared with the epidemic strains in the world.

**Results:**

Among HIV infected patients, the EBV strain variant was mainly EBV-1, while EBV-2 had a higher viral load than EBV-1 (P = 0.001) and EBV-1/2 (P = 0.002). HIV infected patients had higher active virus replication. The EBV LMP-1 variants were mainly the China1 variant. HIV-infected patients had different nucleic acid positions of 30-bp deletion (del30) and had a higher incidence of high 33-bp tandem repeats (rep33) copies than non-HIV-infected patients. There was a difference in the mutations of EBV LMP-1 C-ter del30 and ins15 between HIV infected patients and the control group (P < 0.001).

**Conclusion:**

In southeastern China, EBV in HIV-infected patients had higher active virus replication; EBV infection was mainly EBV-1, and EBV-2 infection has higher EBV virus load; hotspot mutations of LMP-1 C-ter were different between HIV-infected patients and non-HIV-infected patients.

*Trial registration*: This study was approved by the ethics committee of the First Affiliated Hospital of Zhejiang University School of Medicine (Approval No. 2018764), and registered in Chinese Clinical Trial Registry on 3 June 2019 (ChiCTR, ChiCTR1900023600, http://www.chictr.org.cn/usercenter.aspx).

## Background

Epstein-Barr virus (EBV), also known as human herpesvirus 4, infects approximately 95% of people around the world sustaining an asymptomatic life-long infection [1]. EBV is mainly transmitted through saliva [2]. As one of the most common susceptible viruses, EBV induces infectious mononucleosis and is also associated with various lymphoid tumors and epithelial malignancies such as Burkitt's lymphoma, nasopharyngeal carcinoma, gastric cancer, as well as post-transplantation lymphoproliferative diseases [3, 4]. It was demonstrated that EBV-related lymphomas often occur in immunocompromised patients [1]. EBV active infection is common in human immunodeficiency virus (HIV)-infected individuals and may be one of the factors contributing to the pathogenesis of Acquired Immune Deficiency Syndrome (AIDS) [5].

EBV establishes life-long persistent infection in memory B cells and replicates with each cell division [6]. The EBV genome is approximately 170 Kbp long and encodes at least 80 proteins, only some of which have been definitively identified, including six EBV nuclear antigens (EBNA 1, EBNA2, EBNA3A, EBNA3B, EBNA3C, and EBNA-leader protein) and three latent membrane proteins (LMP 1、2A、2B) [7]. Previous studies have shown that there are multiple different variants of EBV [6]. It is widely accepted that EBV can be classified as type 1(EBV-1) and 2 (EBV-2), based on polymorphism in the genes encoding the nuclear antigens EBNA2, EBNA3A, EBNA3B, and EBNA3C. In vitro, the two genotypes exhibit different biological properties, EBV-1 is more efficient than EBV-2 for immortalizing B-cell growth while EBV-2 has increased lytic ability [8, 9]. It was demonstrated that EBV-1 is the predominant variant worldwide, especially in Western and Asian populations, while EBV-2 is more common in Africa [10]. Despite no specific association with disease, it was shown that EBV-2 has a high infection rate in individuals exposed to immunosuppression [11]. With the evolution of molecular biology techniques, they are considered insufficient to describe the entire natural variation of EBV. variations have been described based on polymorphisms of other viral antigens, such as EBNA1 and LPM-1 [12, 13].

LMP-1, an EBV protein with known oncogenic properties, consists of 386 amino acids and is subdivided into 3 domains including a 25 amino acids (aa) N-terminus, six predicted transmembrane domains (aa: 26–196), and a long carboxy-terminal (C-ter) domain (aa: 197–386). Both the N- and C-terminus are located in the cytoplasm of the cell [14]. Relative to B95-8 (accession number V01555.2), LMP-1 sequence variants were classified as China1 (AY337723.1), China2 (AY337724.1), Alaskan (AY337725.1), Med − (AY337721.2), Med + (AY337722.2), North Carolina (NC) (AY337726.2), and China 3 [15]. It has been reported that LMP-1 could promote the growth and inhibit apoptosis of a variety of cell types, exhibiting the characteristics of canonical oncoproteins [16]. Given the biological role of LMP1, the C-ter of LMP-1 was considered essential for its function. If LMP-1-expressing EBV lacks the C-ter of LMP-1, it cannot transduce primary B lymphocytes in vitro [17]. Currently, there are some hotspot mutations in the C-ter of LMP-1 such as the 30-bp deletion (del30), 15-bp insertion (ins15), and 33-bp tandem repeats (rep33). Many studies have shown that these changes may transform EBV into a more aggressive phenotype, increasing its tumorigenicity [18]. For instance, the EBV variants with del30 in LMP-1 C-ter have higher transforming activity, which is more frequently detected in patients with Nasopharyngeal carcinoma [19]. Moreover, a study showed that 100% of patients in Malaysia with peripheral T-cell lymphomas and AIDS-related Hodgkin's lymphoma had been detected with the del30 in LMP-1 C-ter [6]. In addition, according to a study from Argentina, we know that EBV with high numbers of rep33 is easier found among patients with infectious mononuclear cell syndrome and lymphoma than in healthy individuals [20].

It was indicated that del30 in LMP-1 C-ter is distributed differently in various tumors and regions. For example, the incidence of del30 in LMP-1 C-ter was 47.4% in patients with AIDS-related lymphoma in North America, while in Europe it was 53.9%. Among patients with nasopharyngeal carcinoma, the incidence of del30 in LMP-1 in China was 83.8%, compared with 11.1% in North American patients [2]. Most of these studies were concentrated on some neoplastic diseases and healthy populations [16, 21]. However, as an EBV-susceptible population, the biological properties of EBV in HIV patients have not been adequately characterized. Furthermore, it is not clear whether the high prevalence in some EBV variants simply reflects the predominance of these variants in certain geographic regions or due to virus-host interactions leading to enhanced production of LMP1 deletion mutants. In this study, we attempt to demonstrate the characteristics of EBV epidemic strains in patients infected with HIV in southeastern China, including the distribution of EBV genotypes, the level of EBV viral load, and the genetic variability of C-ter of LMP-1.

## Methods

### Patients and samples

This study focused on HIV-infected patients who were hospitalized in the First Affiliated Hospital of Zhejiang University School of Medicine (FAHZU) from February 23, 2018, to July 1, 2019, and was approved by the ethics committee of FAHZU (Approval No. 2018764). HIV infection was defined by the detection of HIV-1 antibodies by an enzyme-linked immunosorbent assay and confirmed by Western blot. EBV infection was defined as EBV DNA detection in plasma. HIV-infected patients with positive EBV DNA were considered to be HIV/EBV co-infection and were included in the study. Furthermore, we also assessed the non-HIV-infected patients who were hospitalized at FAHZU during the same period. We screened the non-HIV infected subjects who had positive plasma EBV DNA and finally enrolled patients into the control group after computerized random sampling and informed consent.

Participants in the study were over the age of 18, of both gender and HIV and/or EBV serology were positive, were not using antiviral therapy, and signed a written consent document. Saliva and whole blood of study participants were collected after signing informed consent. Samples of saliva were collected using a saliva collector and preserved at 4 °C in refrigerator for further use. The whole blood was aliquoted into 2 ml tubes and stored at − 80 °C until use.

### DNA extraction

DNA was extracted from the collected saliva and whole blood using the QIAamp DNA Mini Kit (Qiagen, 40,724 Hilden, Germany) according to the manufacturer’s instruction at a final volume of 200 μL and stored at − 80 °C until further operations.

### EBV viral load determination

The extracted whole blood DNA was used as a template for fluorescence quantitative PCR to determine the EBV viral load. The PCR forward and reverse primers were RTP885-F (5′-GGCCAGAGGTAAGTGGACTTTAAT-3′) and RTP885-R (5′GGGGACCCTGAGACGGG-3′), respectively. The probe sequence was RTP885-P (5′CCCAACACTCCACCACACCCAGGC-3′). The target gene fragment was 96 bp in length. PCR reaction system included the following: 1μL of the DNA template, 5 μL of 2 TaqMan Fast qPCR Master Mix, 0.2 μL each of 10 μmol/L forward and reverse primer, 0.2 μL of 10 μmol/L probe, and 3.4 μL of ddH20. PCR reaction conditions were as follows: pre-denaturation at 94 °C for 3 min; followed by 45 cycles including denaturation at 94 °C for 5 s, annealing at 57 °C for 15 s, and extension at 72 °C for 30 s. The CT value was compared with the standard curve of EBV plasmid XM885-2–1 (2, 788 bp) to obtain the EBV viral load of the patient.

### EBV typing

The extracted saliva DNA was used as the template for PCR in EBV typing. The PCR forward and reverse primers were EBNA3C-F (AGAAGGGGAGCGTGTGTTGT) and EBNA3C-R (GGCTCGTTTTTGACGTCGGC), respectively. Each PCR reaction system included the following: 1 μL of the DNA template, 2.5 μL of 10 × PCR buffer, 2 μL of 25 mmol/L MgCl2, 0.5 μL of 2 mmol/L each dNTP, 1 μL each of the 10 μmol/L forward and reverse primer, 2.5 U of Taq DNA polymerase, and 16.5 μL of ddH20. The PCR reaction conditions were as follows: pre-denaturation at 94 °C for 5 min; followed by 35 cycles including denaturation at 94 °C for 30 s, annealing at 58 °C for 35 s, and extension at 72 °C for 30 s. Then, 5 μL of the PCR product were aliquoted and electrophoresed on a 1% agarose gel for 45 min (electrophoresis parameters: 150 V and 100 mA). Finally, the agar gel was stained with ethidium bromide, and the bands were observed under UV light to determine EBV subtypes. An amplification product of 153 bp for EBV type 1 and a product of 246 bp for EBV type 2.

### EBV LMP-1 C-ter sequencing

The whole blood DNA was used as a template to amplify the C-ter in LMP-1 by semi-nested PCR, and the primers for the first round were LMP1-1F (AAAGGTGTCTGCCAATTCTCG) and LMP1-1R (AGTCACCCTCCTGCTCATCG). Each PCR reaction included the following: 1μL of the DNA template, 2.5 μL of 10 × PCR buffer, 2 μL of 25 mmol/L MgCl2, 0.5 μL of 2 mmol/L dNTP, 1 μL of each 10 μmol/L forward and reverse primer, 2.5 U Taq DNA polymerase, and 16.5μL ddH20. The PCR reaction conditions were as follows: pre-denaturation at 94 °C for 5 min; followed by 35 cycles including denaturation at 94 °C for 30 s, annealing at 58 °C for 35 s, and extension at 72 °C for 1 min. An amplification product of 1, 171-bp was obtained after the first round of PCR, which was subjected to a second round of PCR with primers LMP1-2F (AAGGCATTCCCAGTAAATGGAG) and LMP1-2R (ATTTGCACGGACAGGCATTGTTC). The PCR reaction system and settings were the same as in the first round. If the base peaks obtained by LMP1-2F sequencing overlapped, the primer LMP1-2RCEXU (ACCCCCACTCTGCTCTCAAAACCT) was used for retesting. Finally, an amplification product of 1, 017-bp was obtained. The product was purified using the BigDye Terminator 3.1 kit and sequenced in the ABI 3730 Genetic Analyzer.

### Sequence analysis and phylogenetic tree construction

Sequences were aligned and analyzed with BIOEDIT 7.0.9 software. And LMP-1sequence variants were classified according to the classification scheme introduced by previous studies [22, 23]. In short, seven main international LMP-1 sequence variants, termed China1, China2, China 3 Alaskan, Med − , Med + , and North Carolina (NC), and prototypic LMP-1 (B95.8) were used as reference strains. Sequences obtained from the experiment were compared to the B95.8 reference sequence (accession number V01555.2) and other isolates from the GenBank database. The hotspot mutations of LMP-1 C-ter were analyzed and the phylogenetic tree was calculated with the previously defined evolutionary model using MEGA7.0.2 software [24].

### Data analysis

Data are presented as median (inter-quartile range, IQR) values for continuous variables and numbers (%) for categorical variables. Analysis of categorical variables was based on the χ 2 test or Fisher's Exact test as appropriate. A Mann–Whitney test or Kruskal–Wallis test was used for nonparametric continuous variables. Data of viral load were transformed using the logarithmic function for further analysis of the results. Spearman correlation analysis was used when applicable to examine bivariate relationships. Multivariable analysis using linear regression was used to adjust for significant confounders. All statistical analyses were performed using SPSS 25.0 software with a significance level (α) of 5% and a confidence interval of 95%.

### Nucleotide sequence accession numbers

The nucleotide sequences of LMP-1 C-ter provided herein are in the Genbank database under accession No. MN728815-MN728894 and MT085409-MT085460.

## Results

### Sample Characterization

**Fig. 1:**
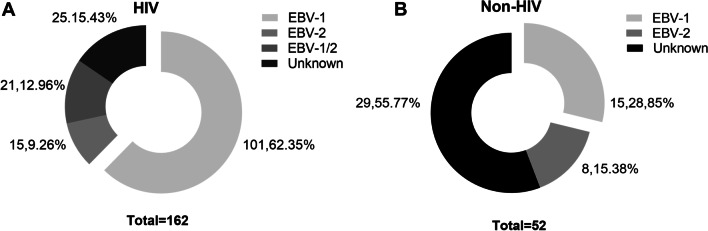
EBV genotype in HIV infected and uninfected patients. **A**: In HIV infected patients, EBV-1 accounted for 62.34% (101/162), EBV-2 accounted for 9.26% (15/162), and co-infecting with EBV-1/2 accounted for 12.96% (21/162). **B**: Among the non-HIV infected individuals, EBV-1 accounted for 28.85% (15/52), and EBV-2 accounted for 15.38% (8/52)

A total of 186 HIV-infected patients who were hospitalized in the First Affiliated Hospital of Zhejiang University School of Medicine were screened between February 23, 2018, and July 1, 2019. 24 (12.9%) subjects were excluded from the study due to the negative plasma EBV DNA at the time of screening. Finally, 162 EBV and HIV-1 coinfected patients were enrolled in the study (male 142, female 20; median age 47.5 y [range 21–81 y]). Among non-HIV patients, a total of 52 subjects met the inclusion criteria and were therefore included in the analyses of this article (male 31, female 21; median age 53.5 y [range 18–90 y]) (Table 1). EBV genotype was identified from saliva in 84.57% (137/162) of HIV infected patients (Fig. 1A). Among those HIV/EBV coinfected population, the EBV-1was the most prevalent (101/162, 62.35%) genotype, followed by co-infection with both genotypes (21/162, 12.96%) and the EBV-2 genotype (15/162, 9.26%) (Fig. 1B). However, in the individuals with EBV infection alone, only 23 of 52 (44.23%) cases confirmed the EBV genotype, among which EBV-1accounted for 28.85% (15/52) and EBV type 2 accounted for 15.38% (8/52). Compare to HIV negative participants, the frequency of co-infecting with EBV-1/2 was higher in HIV infected patients (P = 0.001).

**Table 1:** Description of patients with HIV-1 and the control group

Category	HIV (N = 162)	Non-HIV (N = 52)	*P*-value
*Age*	47.5 (35.9, 58.9)	53.5 (44.0, 68.5)	0.018
*Gender*			0.001
Male	142 (87.65)	31 (59.62)	
Female	20 (12.35)	21 (40.38)	
*Education*			0.001
Primary school	43 (26.54)	32 (61.54)	
Secondary school	83 (51.23)	15 (28.85)	
Undergraduate or above	36 (22.22)	5 (9.62)	
*Comorbidities*			
Lymphomas	38 (23.46)	17 (32.69)	0.204
Diseases other than lymphoma	124 (76.54)	35 (67.31)	
*HAART*			
INSTI-based regimens	78 (48.15)	–	
PI-based regimens	5 (3.09)	–	
NRTI-based regimens	79 (48.77)	–	
*HAART exposure duration*			
≤ 6 months	116	–	
6–12 months	42	–	
12–24 months	2	–	
≥ 24 months	2	–	
*CD4*^+^ *T cell count(cells/mm*^*3*^*)*	96.50 (21.75–241.50)	–	
*CD8* + *T cell count(cells/mm*^*3*^*)*	455.50 (250.25–750.00)	–	
*CD4*^+^*/CD8*^+^	0.19 (0.08–0.47)	–	

Data was are median (IQR) or n (%); HAART: highly active antiretroviral therapy, NRTIs: Nucleotide Reverse Transcriptase Inhibitors, NNRTIs: Non-nucleotide Reverse Transcriptase Inhibitors, INSTIs: Integrase strand transfer inhibitors

### Differential EBV viral load in HIV-infected and HIV-uninfected patients

**Fig. 2:**
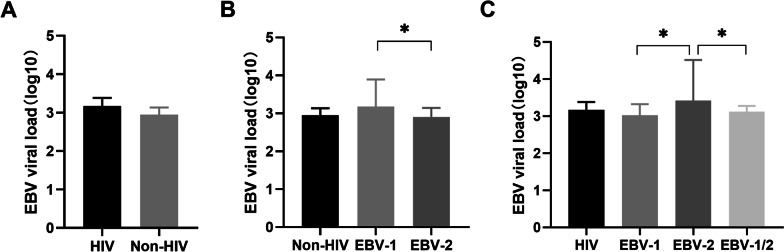
EBV viral load of patients with HIV and the control group. **A**: EBV viral load levels in patients classified according to HIV infection. **B**: EBV viral load levels in HIV negative patients classified according to EBV genotype. **C** EBV viral load levels in HIV positive patients classified according to EBV genotype. *P < 0.05

In this study, EBV viral load was compared between the HIV and non-HIV groups (Fig. 2). The median log10 of EBV viral load was 3.17 copies/ul (IQR: 2.67–3.38) in the HIV positive group, while 2.95 (IQR: 2.84,3.13) in HIV negative group. In an intergroup analysis, the median plasma viral load in the HIV group was higher (intergroup difference: 0.14 95%CI -0.02, 0.27) but not statistically significant (P = 0.093). In the multiple linear regression analysis with adjustment for potential confounding variables, we did not observe a correlation between EBV viral load and HIV infection. However, it was found that there was a difference in EBV viral load levels of different subtypes of EBV for both groups. In HIV uninfected individuals, the median log10 viral load in EBV-1genotype (median: 3.18 IQR 3.00–3.90) was higher compared with the EBV-2 genotype infected patients (median: 2.91 IQR 2.79–3.14, intergroup difference: 0.28 95%CI 0.04, 0.89, P = 0.047). In HIV infected patients, the EBV-2 group shows a higher level of viral load when compared to others [P = 0.001 (EBV-2: median 3.42 IQR 3.29–4.51) (EBV-1: median: 3.02 IQR 2.61–3.32) (EBV-1/2: 3.12 IQR 2.42 3.28)] (Fig. 2). In addition, we further evaluated the correlation between EBV viral and host immunological profiles. CD4 + T lymphocytes and CD8 + T lymphocytes were observed positive correlation with EBV viral load, which was also consistent in HIV/EBV-1 and HIV/EBV-1/2 groups for based subgroup analysis based on EBV genotype. Nevertheless, there was no relationship between the EBV viral load and HAART exposure duration.

### LMP1 variant characterization in HIV and non-HIV infected patients

**Fig. 3:**
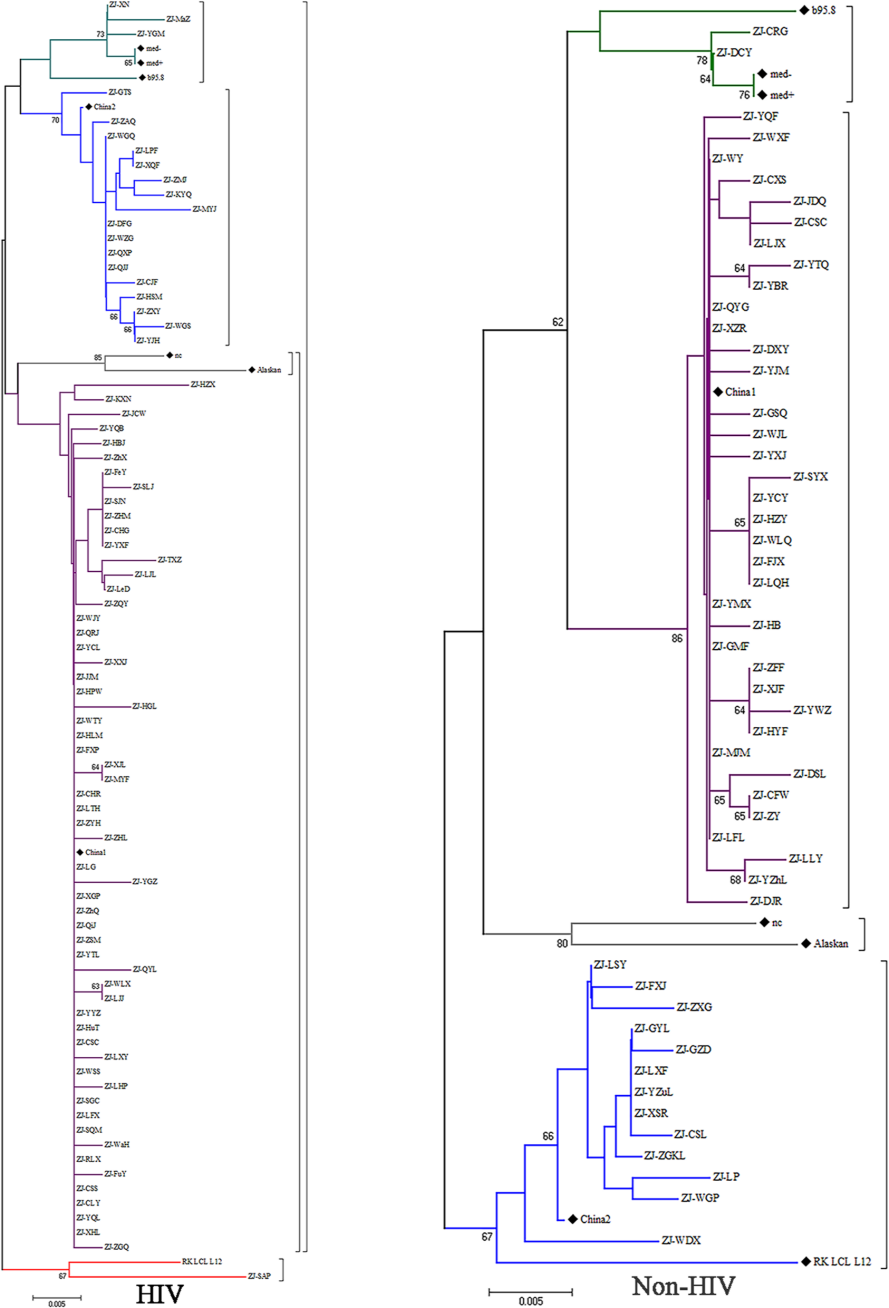
Phylogenetic tree of EBV LMP-1 C-ter of HIV positive and HIV negative group. ♦: Eight reference sequences (b95.8, China1, China2, med + , med-, Alaskan, nc,RK_LCL_L12(MG298910.1)).The tree was rooted by middle point method, bootstrap values were obtained after 1000 resamplings, and only bootstrap values over 60% were shown. Purple means China1 clade, gray means nc/Alaskan clade, green means med + /med- clade, blue means China2 clade

In HIV-infected patients and HIV negative individuals, a total of 80 and 52 LMP-1 C-ter nucleotide sequences were obtained and translated into amino acids for the phylogenetic analysis. The C-ter region of the LMP1 gene from patients and reference sequences from GenBank were used for phylogenetic tree reconstructions (Fig. 3). The China 1 variant is most prevalent in southeastern China, both among HIV-infected and non-HIV-infected individuals. Among the HIV infected patients, the China1 variants was the most prevalent (59/80, 73.75%), followed by China 2 (17/80, 21.25%) and Med + /Med- (3/80, 3.75%). Of note, the remaining one patient exhibited a so distinctive LMP-ter sequence from the reference sequence that it could not be identified. A similar LMP1 variant characterization was observed in HIV negative individuals. China 1 variant was detected in 37/52 (71.15%) of the individuals, followed by China 2 in 13 (25.00%) individuals and Med + /Med- in 2 individuals (3.85%). No statistically significant difference was identified in the LMP1 variant distribution between the two groups (Table 2).

**Table 2:** The hot mutations of LMP-1 C-ter in patients with HIV and the control group

	HIV-infected patients (n = 80)	HIV-negative participants(n = 52)	P value
LMP1 variant characterization			0.831
China 1	59 (73.75)	37 (71.15)	
China 2	17 (21.25)	13 (25.00)	
China 3	0 (0)	0 (0)	
Med + /Med-	3 (3.75)	2 (3.85)	
Alaskan	0 (0)	0 (0)	
North Carolina	0 (0)	0 (0)	
Unknown	1 (1.25)	0 (0)	
LMP1 variant distribution			
del30 present	59 (73.75)	37 (71.15)	0.743
Position in B95-8		< 0.001	
168,257–168,286	43 (53.75)	0 (0)	
168,266–168,295	14 (17.50)	37 (71.75)	
168,267–168,296	1 (1.25)	0 (0)	
168,242–168,340	1 (1.25)	0 (0)	
None	21 (26.25)	15 (28.85)	
ins15 present	80 (100)	17 (32.69)	< 0.0001
Position in B95-8			
168,400–168,414	80 (100%)	17 (32.69)	
None	0 (0)	35 (67.71)	
No. of rep33 units			0.675
2.5copies	2 (2.50)	4 (7.69)	
3.5copies	19 (23.75)	12 (23.08)	
4.5copies	21 (26.25)	12 (23.08)	
5.5copies	18 (22.25)	16 (30.77)	
6.5copies	13 (16.25)	7 (13.46)	
7.5copies	4 (5.00)	1 (1.92)	
8.5copies	2 (2.50)	0 (0)	
9.5copies	1 (1.25)	0 (0)	

Data was are median (IQR) or n (%); LMP-1: latent membrane protein 1, C-ter: carboxy terminus, HIV: human immunodeficiency virus, del30: 30-bp deletion, ins15: 15-bp insertion, rep33:33-bp tandem repeats

The LMP variants and gene polymorphisms in HIV infected patients and non-HIV patients are described in Table 2. It was observed that there was no statistically significant difference in the del30 incidence of LMPs between HIV-infected patients and controls, with del30 detected in 59 (73.75%) HIV positive persons, and 37(71.15%) HIV negative persons, respectively. However, upon further analysis of the distribution of the 30 bp deletion, we found the position of del30 between the two groups is different. Among HIV infected patients, the strains with del30 were all China1 variants and the nucleotide position was located in 168,286–168,257, 168,295–168,266, 168,296–168,267, 168,340–168,242 respectively. While in HIV negative individuals, the position of del30 is relatively fixed, located in 168,295–168,266. In addition, ins 15 was present in greater frequencies in HIV-positive compared with HIV-negative participants (P < 0.001). Ins15 (168,414–168,400) occurred in all 80 (100%) LMP-1 C-ter strains in HIV infected patients while only 17 cases (17/52, 32.69%) in HIV-negative patients. Furthermore, according to the copy number of rep33, the sample was divided into 2 groups: low (≤ 4.5 copies) and high (≥ 5.5 copies) numbers of rep33 units. In this way, the copy number of rep33 was not found to be statistically different between the two groups (P > 0.05). Nevertheless, in HIV infected patients several cases with a higher copy number of rep33 had been observed, including a 8.5 and a 9.5 rep33 units (Table 2). Moreover, we attempted to explore any possible association between different polymorphisms in the LMP-1 C-ter by analyzing samples obtained from both groups. A significant correlation was found between the presence of a high copies number of rep33 and del30 (r = 0.44, P = 0.001), ins15 (r = − 0.26, P = 0.003). However, the association between polymorphisms in the LMP-1 C-ter and EBV viral load was not significant (P > 0.05).

## Discussion

EBV, a lymphotropic human gamma-1 herpes virus, is a common cause of infection in humans worldwide [5]. It was generally accepted that EBV could be divided into two types, EBV-1 and EBV-2, distinguished based on genetic differences in the EBNA2 and EBNA3 genes [6]. Over the last decade, considering variation in the EBV genome, several EBV variants had been introduced. Researchers have tried to clarify whether these variants are associated with racial groups, geographic regions, or diseases. Although the EBV strain variation has been intensively studied in several neoplasms in different geographic regions [25], as an EBV-susceptible population, the knowledge of EBV strain variation in HIV infected patients has remained scarce. In this cross-sectional study, we characterized EBV polymorphisms at EBNA3C gene and LMP-1 C-ter in HIV positive patients and HIV negative individuals in Southeast China.

In this study, it was indicated that the EBV subtypes in 214 saliva samples were predominantly EBV-1 for HIV positive and negative individuals in southeastern China, which was consistent with that reported previously [11]. In addition, several studies have found that EBV-2 or co-infection is more frequently found in immunosuppressed patients indicating that host *immune* function could influence the reactivation or latency of EBV infection [9, 10]. Agreeing with these authors, a higher incidence of EBV-1/2 was found in HIV-infected patients compared to HIV- negative individuals. However, compared to South America (45/138, 32.61%), a lower frequency of EBV-1/2 in HIV infected patients in coastal China was reported in our study, which is only 12.96% (21/162). This may be a reflection of the distribution characteristics of EBV variants in southeastern China. In vitro, different subtypes of EBV exhibit variable biological properties. EBV-1 is more conducive to the growth of immortalized B lymphocytes, whereas EBV-2 shows a strong lytic capacity [13]. It was believed that EBV-1 was more likely to induce infectious mononucleosis than EBV-2 [26], but a subsequent study found no significant difference [27]. In addition, subtype variants of EBNA2 have been studied in EBV-associated carcinoma in China, but there is little evidence of its association with disease [28].

Furthermore, a higher EBV viral load in HIV positive group was found in our study, although the difference was not statistically significant. It was demonstrated that EBV viral load could be a biomarker of active and replicative infection because of few free viral particles circulating in plasma [29]. Patients with high EBV DNA load had higher levels of pro-inflammatory cytokines (IL-6, IL-10, TNF-α) than those with low EBV levels, and EBV load was strongly correlated with the percentage of activated B cells [30]. Moreover, it has been reported that patients infected with HIV with high EBV viral load have an increased risk of developing B lymphoma [31]. And HIV patients tend to have higher baseline levels of circulating EBV than healthy individuals [30]. Besides, the level of EBV viral load is apparently related to the degree of immunosuppression [32]. In our study, however, a positive correlation between CD4 + T cells and EBV viral load was observed. On the one hand, these seemingly inconsistent findings might be attributed to the relatively small sample sizes in our study, and on the other hand, it was believed to be related to the confounding factor of HIV viral load. It was indicated that EBV viral load could be higher in patients with detectable HIV plasma viremia than in those with undetectable plasma viremia, despite their favorable immune status (CD4 > 500 cells/μl) [30].

It was observed in our study that different EBV subtypes showed a different levels of EBV viral load (*P* = 0.001). A higher viral load of EBV-2 was shown in HIV positive participants while lower than EBV-1 in HIV negative group. It was reported in EBV-2/HIV coinfected patients, EBV-2 viral load was associated with low plasma levels of HIV viral load, with trends toward the maintenance of the CD4 + and CD8 + T lymphocytes [33]. As was demonstrated in co-infection, EBV-2 modulates the immune response, leading to an anti-inflammatory microenvironment, which eventually impedes the overall inflammatory state of the progression of HIV infection [34, 35]. Of note, however, in the HIV/EBV-1 co-infected group, EBV viral load was positively correlated with some pro-inflammatory factors such as IL-6, and IL-10 [33]. The relationship between EBV genotypes and immunopathological profile is particularly interesting to explore further.

LMP-1 is an important functional protein in EBV oncogenicity, for its ability to induce phenotypic changes in both B-cells and epithelial cells [32]. And according to the variability of the C-terminus region of LMP-1, EBV was classified as multiple LMP1 variants, in which China 1 was previously reported to be the most prevalent LMP1 variant in Asia [10]. In our study, we found that China 1 was the predominant LMP1 variant in both HIV-positive and negative individuals, followed by China 2 and Med + /Med-. Studies have reported that the China1, Alaskan and Med + variants can enhance the NF-κB signaling pathway, and help B lymphocytes to escape apoptosis [36]. NF-κB can directly or indirectly mediate the upregulation of many LMP-1-induced genes, which encode ICAM-1, CD40, IL-6, LFA-3, A20, Fas, and TRAF1 [37]. ICAM-1 and LFA-3, involves in cell adhesion, and the expression of A20, CD40, and TRAF1 can prevent the apoptosis of B lymphocytes. IL-6 may limit the response of NK and CTL to EBV infection to avoid immunosurveillance. NF-κB plays a pivotal role in the prevention of apoptosis of EBV-transduced B lymphocytes and fibroblast transformation [37, 38]. In addition, a higher incidence of del30 was observed in the HIV infected patients in southeastern China was observed. The incidence of del30 was 73.75% (59/80), and all of the 59 variants were of the China1 variant. It has been reported that the China1 and Med + variants have 30-bp deletion [16]. In lymphoma and nasopharyngeal carcinoma, EBV strains with LMP-1 del30 mutation have higher tumorigenicity than strains without deletion [39], which enhances the transduction ability of EBV without affecting the NF-κB signaling pathway [36]. It has been reported that del30 increases the half-life of LMP-1 from 3 to 24 h [39]. Besides, the nucleotide position of del30 was different in HIV patients, which has not previously been reported and warrants further investigation. All LMP-1 C-ter strains had rep33 but the number of copies was different between the two groups. Despite the lack of a statistically significant difference, HIV patients had higher copies of rep33 than the control group. As a study reported, a high copy number of rep33 is associated with the malignancy of EBV [2]. Whether del30 at different locations and the number of copies of rep33 will affect the tumorigenicity of EBV strains are to be verified with larger sample sizes in the future.

There are several limitations to this work. First, as a cross-sectional study based on real-world data, the study is limited by a lack of a valid control group. In our study, there was an imbalance in age, gender, etc. between the HIV-positive and control groups. Despite adjustment for multiple confounders, the risk of residual unmeasured confounding remains possible. we could only describe the EBV variation and clinical relationship identified in this study, which worth to be further investigating using other study designs. Second, due to the relatively small size of samples, the EBV genotype was identified with a relatively low positive rate in HIV negative group compared with HIV positive group. The better assays in a larger sample size require further exploration.

## Conclusions

In conclusion, this study described the EBV molecular epidemiology and investigated the association between EBV genotypes and the immunological profiles in people living with HIV in southeastern China. EBV-1 was the predominant EBV strain in HIV infected patients and China1 (LMP-1) variants were the most prevalent variants.

A relatively high incidence of EBV-1/2 was found in HIV-infected patients compared to HIV- negative individuals. EBV in HIV-infected patients had higher active virus replication and EBV-2 infection had a higher EBV virus load. Compared with non-HIV-infected patients, HIV patients had unique characteristics in the del30, ins15 and rep33 hotspot mutations in the LMP-1 variant. These observations highlight the importance of the follow-up of HIV infected patients within a few years to monitor the possible involvement of EBV in oncogenicity and also to establish whether del30, ins 15, and a high number of rep33 copies, truly represent an increased risk for EBV-associated malignancies in our geographical region.

## Data Availability

The datasets used and/or analysed during the current study are available from the corresponding author on reasonable request.

## Abbreviations

EBV: Epstein-Barr virus
LMP-1: Latent membrane protein 1
C-ter: Carboxy terminus
del30: 30-Bp deletion
ins15: 15-Bp insertion
rep33: 33-Bp tandem repeats
AIDS: Acquired immune deficiency syndrome
